# Efficacy of a single ultrasound-guided injection of high molecular weight hyaluronic acid combined with collagen tripeptide in patients with knee osteoarthritis and chondrocalcinosis

**DOI:** 10.3389/fmed.2024.1437160

**Published:** 2024-07-19

**Authors:** Francesco Porta, Emilio Filippucci, Edoardo Cipolletta, Marco La Grua, Xenia Barni, Silvia Sirotti, Florentin Ananu Vreju

**Affiliations:** ^1^Interdisciplinary Pain Medicine Unit, Santa Maria Maddalena Hospital, Occhiobello, Italy; ^2^Rheumatology Unit, Department of Clinical and Molecular Sciences, Polytechnic University of Marche, Ancona, Italy; ^3^Academic Rheumatology, University of Nottingham, Nottingham, United Kingdom; ^4^Department of Rheumatology, IRCCS Ospedale Galeazzi-Sant’Ambrogio, Milan, Italy; ^5^Department of Rheumatology, University of Medicine and Pharmacy Craiova, Craiova, Romania

**Keywords:** osteoarthritis, CPPD, hyaluronic acid, collagen tripeptide, ultrasound guided joint injection, WOMAC

## Abstract

**Introduction:**

Osteoarthritis (OA) and calcium pyrophosphate deposition (CPPD) often co-exist, this resulting in a clinical condition characterized by amplified inflammation and more severe and faster cartilage degeneration compared to OA alone. Our study aims to explore the efficacy of a therapeutic approach that addresses both conditions, using a combination of a high molecular weight hyaluronic acid (HMWHA) and collagen tripeptide (CTP). Additionally, safety profile and baseline characteristic predictive value were evaluated.

**Methods:**

We conducted a retrospective study on patients diagnosed with symptomatic knee OA (KOA) and CPPD treated by ultrasound (US) guided intraarticular injections of HMWHA-CT in the outpatient clinics of the Interdisciplinary Pain Medicine Unit at Santa Maria Maddalena Hospital, Occhiobello, Italy and in the Rheumatology Unit of the Emergency County Hospital Craiova, Romania (ECH Craiova). All the patients underwent clinical and US evaluation at baseline, 1, 3, and 6 months. From clinical point of view, Numeric Rating Scale (NRS) pain and Western Ontario and McMaster Universities Osteoarthritis Index (WOMAC) were recorded. US data included detection of synovitis, cartilage damage, osteophytes, and CPPD deposits. Clinical efficacy was defined with NRS and WOMAC variations in respect to baseline and using the minimal clinically important difference values: an improvement of 2 point for NRS pain and 10 for the total score for WOMAC.

**Results:**

Twenty-nine patients (34 knees) were injected and evaluated. Overall pain levels, as measured by NRS, demonstrated a consistent decrease in patients across all follow-up intervals, with the most substantial improvement at the 6-month compared to baseline measurements. A significative proportion of patients achieved the minimum clinically detectable improvement, specifically 79% for NRS and 83% for WOMAC (19 and 20 patients, respectively).

**Conclusion:**

Our data showed a significant efficacy of ultrasound guided HMWHA-CT, in patients with KOA and CPPD, thus making it reasonable to consider that the combination of HMWHA and CTP can provide a strong anti-inflammatory effect.

## Background

Osteoarthritis (OA) is one of the most common chronic arthropathies, presenting with joint pain, functional impairment, and progressive cartilage degeneration (1). Despite its high prevalence, existing treatments are primarily focused only on pain reduction and limiting joint impairment. Therapeutic strategies supported by the current scientific literature include the use of food supplements, paracetamol, anti-inflammatory drugs, hyaluronic acid or corticosteroid injections, physiotherapy, regular physical activity, maintaining an appropriate body weight to reduce stress on the joints, and in advanced disease, prosthetic joint replacement (2).

On the other hand, calcium pyrophosphate deposition (CPPD) disease emerges as a chronic joint condition caused by the accumulation of calcium pyrophosphate crystals in the joint cartilage, leading to inflammatory stimuli and cartilage damage (3). The therapeutic approach most supported by scientific literature involves the use of colchicine as the first-line treatment for managing the acute inflammatory phase. However, there are no therapeutic strategies for managing the non-acute phases (4) except some data restricted to retrospective case-analysis about the off-label use of anti-inflammatory and immunomodulant drugs (5).

The coexistence of OA and CPPD results in a clinical condition characterized by amplified inflammation and faster and more severe cartilage degeneration compared to OA alone (6–8). Despite the extensive literature on specific treatments for OA, there is currently no standardized therapeutic approach to address the combination of OA and CPPD (6). This gap becomes even more relevant considering the prevalence of both conditions in the elderly population (9), often unsuitable to surgical procedures, like prosthetic joint replacement, but requiring clinical management for symptom resolution (10).

Our study aimed to explore the efficacy of a single intra-articular injection of high molecular weight hyaluronic acid (HMWHA) and collagen tripeptide (CTP) in patients with knee OA (KOA) with imaging evidence of cartilage calcification/chondrocalcinosis. HMWHA is known for its pain-reducing and functional improvement capabilities (11, 12), and CTP is known for its anti-inflammatory and regenerative properties (13). “*Chondroplus*” is a pharmaceutical product that combines both agents and theoretically exerts its biological activity up to 1 year (14–17) thus it can be a valuable option for CPPD concurrent with OA.

The secondary aims of this study were to evaluate the safety of Chondroplus in patients with KOA and CPPD, and to identify baseline clinical and US predictors of achieving a minimum clinically detectable improvement (MCDI) in the pain score 6-month after the injection.

## Materials and methods

This is a retrospective study involving patients with symptomatic KOA with CPPD (18) treated with intraarticular injections of HMWHA-CTP in the outpatient clinics of the Interdisciplinary Pain Medicine Unit at Santa Maria Maddalena Hospital, Occhiobello, Italy and in the Rheumatology Unit of the Emergency County Hospital Craiova, Romania (ECH Craiova).

Patients were considered eligible for inclusion if they met all the following criteria: 18 years of age or older, fulfillment of the American College of Rheumatology (ACR) classification criteria for KOA (12), at least one episode of clinically reported knee inflammatory flare, presence of KOA on conventional radiograph (any grade), presence of CPPD at X-rays or US according to previously published definitions (19, 20) and with a NRS knee pain ≥3 (0–10) before the injection.

The exclusion criteria included a known diagnosis of inflammatory joint disease, other than CPPD disease, previous intra-articular injection within 6 months and the impossibility or refusal to sign the informed consent for publication.

Our usual clinical practice includes a strict follow-up in case of injections in patients with complicated KOA or when a relatively new device is used. According to this, all the patients that undergo such procedure are evaluated four times: during the first injection of HMWHA-CTP (baseline visit, T0), 1, 3, and 6 months after baseline visit (second, third, and fourth follow-up visits, T1–T2–T3). After 2 weeks from the injection patients receive a phone call to ask for adverse event and to record pain-NRS.

During baseline visit, the following clinical data are recorded: demographic data (age and gender), anthropometric data (height and weight), previous or concomitant pharmacologic treatment, patients-reported outcomes (PROs) [NRS pain (0–10) and Western Ontario and McMaster Universities (WOMAC)]. WOMAC and NRS pain were recorded also during any follow-up.

At baseline and T3 the following ultrasonographic data are recorded: semi-quantitative (0–3) assessment of articular fluid collection, synovial hypertrophy (SH), power Doppler (PD) signal, CPPD absence/presence, presence and grading of osteophytes, meniscal protrusion (dichotomous score), and cartilage damage (semiquantitative score) (21, 22) (Table 1).

**TABLE 1:** Baseline characteristics of enrolled patients.

Baseline characteristics	Mean (SD) or absolute frequency and relative percentage
Age (years)	70.3 (12.4)
BMI	26.9 (4.2)
Female sex	12 (35.3)
**K-L grade**	
2	6 (19.4)
3	10 (32.3)
4	15 (48.4)
Failed former therapies	11 (32.4)
NRS	5.4 (1.9)
WOMAC	86.1 (47.4)
WOMAC pain	17.1 (8.8)
WOMAC stiffness	7.3 (4.6)
WOMAC function	61.8 (37.1)
Joint effusion at US assessment	25 (86.2)
Synovial hypertrophy at US assessment	27 (79.4)
Synovial vascularization at US assessment	6 (23.1)
Meniscal protrusion >3 mm at US assessment	13 (52.0)
Meniscal protrusion (mm) at US assessment	4.1 (3.3)
Cartilage damage at US assessment	23 (67.7)
Osteophytes at US assessment	23 (67.7)

BMI, body mass index; K-L, Kellgren-Lawrence; NRS, Numeric Rating Scale; WOMAC, Western Ontario and McMaster Universities Osteoarthritis Index; US, ultrasonography.

For patients taking medications, any change in painkiller or anti-inflammatory drugs intake was recorded.

During patients’ selection we excluded those with missing follow-up data. Specifically we excluded patients that did not present at T1 or T2 follow-up.

Clinical data were recorded by two rheumatologists (FP and FV) and a trained nurse (XB). All the ultrasound (US) evaluations and the US-guided injections were performed by two rheumatologists (FP and FV) with more than 15 years of experience in the field of diagnostic and interventional US. The study was approved by the local ethics committee of ECH Craiova (No. 22763/09.05.2023) and conducted in accordance with the Helsinki Declaration and all patients gave their informed consent to enrolment.

### Ultrasound examination

Ultrasound was carried out to identify the presence of CPPD, to define the inflammatory and structural alterations at baseline and during follow-up visits and to guide injections.

Patients had their knees scanned with ECube 12 US system equipped with a 6–17 MHz linear probe (Alpinion Medical Systems, Seoul, Republic of Korea) or MyLab X8 US system equipped with a 9–15 MHz linear probe (Esaote SpA, Genoa, Italy), according to the 2017 EULAR standardized procedures for US imaging (23). US assessment was aimed at detecting fluid collection, s SH, PD signal, CPPD deposits, presence and grade (0–3) of osteophytes, meniscal protrusion (millimeters from joint line in neutral position and not weight bearing), and cartilage damage.

Synovial hypertrophy was defined as the presence of abnormal hypoechoic synovial tissue within the capsule that is not displaceable and poorly compressible, and may exhibit power PD signal, while synovial effusion (SE) is defined as abnormal hypoechoic or anechoic intra-articular material that is displaceable and compressible but does not exhibit a Doppler signal (21). Capsular distention was graded according to a 0–3 semi-quantitative score for these components: grade 0 = absent; grade 1 (mild) = small hypoechoic/anechoic line beneath the joint capsule; grade 2 (moderate) = capsule elevated parallel to the joint area; and grade 3 (severe) = strong convex distension of the joint (24). Such scoring system was applied to images acquired using the longitudinal suprapatellar scan.

As regards Doppler signal, the following grading system especially developed for large joint was adopted: grade 0 = absence of intra-articular Doppler signal; grade 1 (mild) = presence of up to three single Doppler signals or two single Doppler signals and one confluent Doppler signal representing only low flow; grade 2 (moderate) = grade higher than grade 1 and with less than 50% of the intra-articular area filled with Doppler signals representing clear flow; and grade 3 (severe) = with more than 50% of the intra-articular area filled with Doppler signals (24).

Cartilage damage was defined as the loss of anechoic structure and/or thinning of the cartilage layer, and irregularities and/or loss of sharpness of at least one cartilage margin. Grading of cartilage damage is classified on a 0–3 semi-quantitative score (grade 0 = normal cartilage; grade 1 (mild) = loss of anechoic structure and/or focal thinning of the cartilage layer OR irregularities and/or loss of sharpness of at least one cartilage margin; grade 2 (moderate) = loss of anechoic structure and/or focal thinning of the cartilage layer AND irregularities and/or loss of sharpness of at least one cartilage margin; and grade 3 (severe) = focal absence or complete loss of the cartilage layer (21).

Osteophytes were defined as step-up bony prominences at the articular surface margin visible in at least two perpendicular planes. According to the OMERACT criteria, they were graded on a 0–3 semiquantitative score (0 = absence of osteophytes, 1 = small beak-like osteophyte, 2 = intermediate-sized osteophytes, 3 = proliferative or mushroom-sized osteophytes) (22).

Calcium pyrophosphate deposition was characterized by the presence of hyperechoic deposits, which could vary in size and shape, could be detected within the fibrocartilage structure and/or the hyaline cartilage. These deposits either stay fixed or move with the joint during dynamic evaluations. CPPD deposits were evaluated as either absent (0) or present (1) (25).

Meniscal protrusion was defined as a displacement of the meniscal body with respect to the margin of the tibial plateau ≥3 mm. Through US assessment, we determined the extent of the meniscal protrusion in millimeters (26).

### Ultrasound-guided injection

All patients underwent one US-guided injection with Chondroplus at baseline.

**FIGURE 1:**
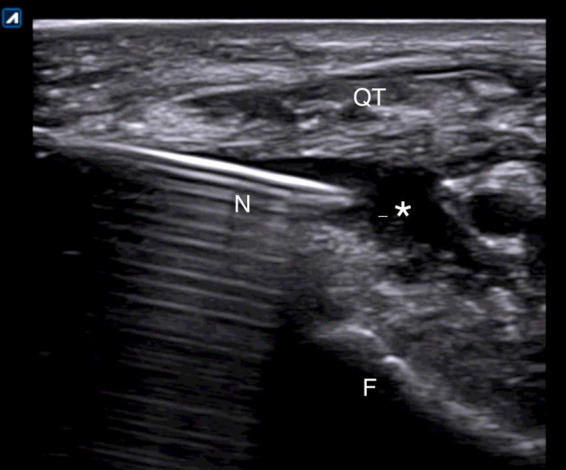
Suprapatellar lateral approach for in-plane US-guided knee injection in presence of effusion. The needle is entering the joint just below the quadriceps tendon. N, needle; QT, quadriceps tendon; F, femoral bone; *, effusion.

**FIGURE 2:**
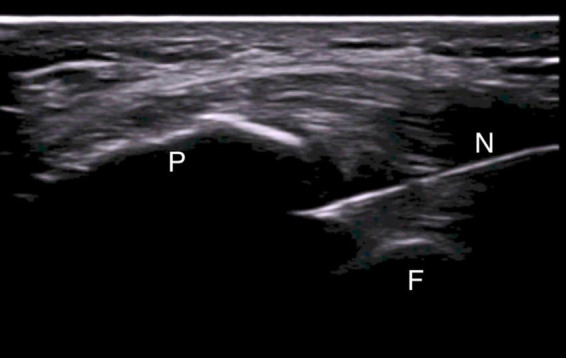
Mid-patellar lateral approach for in-plane US-guided knee injection in absence of effusion. The needle is entering the joint between the patella and the femoral bone. N, needle; P, patella; F, femoral bone.

Ultrasound-guided injections were performed according to standardized protocol (27). All patients were asked to lay down on the bed with the leg flexed about 20° sustained with a pillow under the popliteal area. A direct US-guided in-plane approach to visualize the correct needle tip placement was used. In case of effusion, a suprapatellar latero-medial approach putting the probe in transverse view in respect to the knee was used and the effusion was aspirated before the release of the drug (Figure 1). In case of absence of effusion, a lateral midpatellar approach was preferred for the injection, with an inplane approach for the needle visualization (Figure 2).

The correctness of the procedure, defined as right needle positioning and the spreading of the drug, was verified by real-time US assessment.

All the injections have been performed in sterile conditions using a 21G needle.

### Outcome measurements

Primary outcome measure was the NRS pain. Secondary outcomes were Western Ontario and McMaster Universities Osteoarthritis Index (WOMAC) total score and subscales and US features reflecting joint inflammation (joint effusion, SH, and PD signal).

The WOMAC is an OA-specific disease assessment scale that is regularly used in clinical trials. It consists of 24 items divided into three subscales: Pain (5 items), Stiffness (2 items), and Physical Function (17 items), each assessed using a 0–10 NRS, where 0 indicates no pain/stiffness/difficulty and 10 indicates extreme pain/stiffness/difficulty. Patients were required to complete the questionnaire regarding the previous 48-h recall period. Scores for total WOMAC and each subdomain were calculated from the average score of the component questions and higher score indicating worse outcomes (28, 29).

Clinical efficacy of injection was defined using the minimal clinically important difference values as an improvement of 2 point for NRS pain (30) and of 11 points for pain, 8 for stiffness, 9 for function, and 10 for the total score for WOMAC (31).

Ultrasound findings reflecting inflammation were used as secondary outcome measure tool in order to evaluate the anti-inflammatory effect of collagen and hyaluronic acid (15, 32).

Finally, every side effect possibly related to the injections was recorded with particular attention to post-injection flares.

### Statistical analysis

Descriptive statistics were presented with absolute and relative frequencies or mean and standard deviation, as appropriate.

The mean change of NRS and WOMAC at follow-up has been evaluated with one-way ANOVA.

To identify any predictors of treatment response, first, we compared patients with and without a significant improvement in the NRS after 6 months using univariable logistic models. Then, we included all the predictors with a *p*-value < 0.10 at the univariable analysis in a multivariable logistic model.

The significance value was set at 0.05. Statistical analyses were carried out using STATA SE v18.0 (STATACorp, College Station, TX, USA).

## Results

Twenty-nine patients [12 women, mean age 70.3 years (12.4 SD), body mass index (BMI) 26.9 (4.2 SD), median K-L of 3], including a total of 34 knees affected by OA graded 2–4, and CPPD, underwent intra-articular injection with Chondroplus (2 ml) and had a follow-up of at least 3 months; 6 months follow-up was also available in 25 of these patients (86.2%).

The majority of patients (25/29, 86.2%) lacked prior traumatic injury history, while 20% had previously undergone unsuccessful treatments with hyaluronic acid or platelet-rich plasma (PRP).

Only two patients were not included: the first did not come at T2 and presented at T3 follow-up visit and the second returned after 10 months. Both the patients did not present due to improved clinical wellbeing and did not report any side effects.

All baseline data are summarized in Table 1.

### Variation of NRS and WOMAC in respect to baseline

**FIGURE 3:**
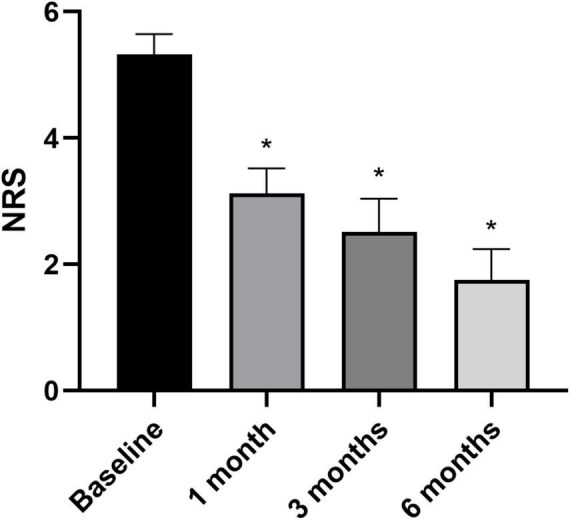
Pain assessed through Numeric Rating Scale at baseline (*n* = 34), 1 month (*n* = 34), 3 months (*n* = 34), and 6 months (*n* = 24). *p < 0.05 vs. baseline. *N* refers to the number of knees.

**FIGURE 4:**
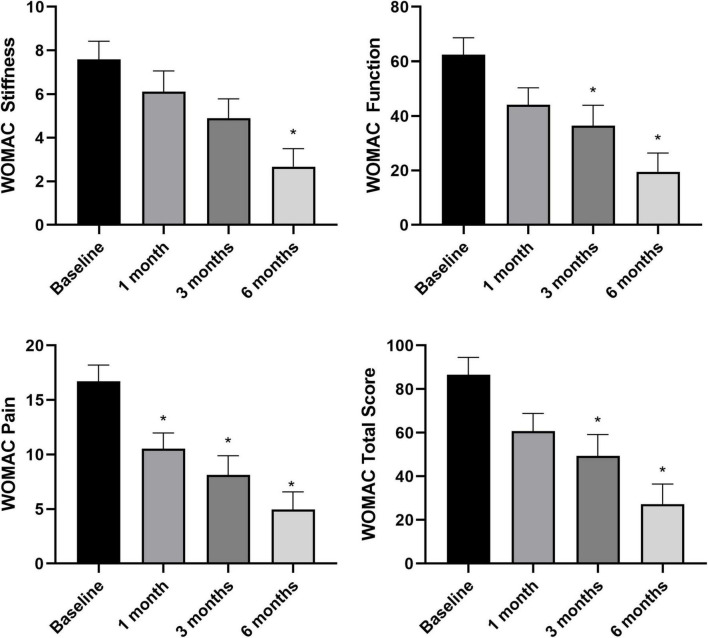
Knee functionality assessed through WOMAC sub-scales and total score at baseline (*n* = 34), 1 month (*n* = 34), 3 months (*n* = 34), and 6 months (*n* = 24). *p < 0.05 vs. baseline. *N* refers to the number of knees.

Overall pain levels, as assessed through the NRS, demonstrated a consistent decrease in patients across all follow-up intervals, with statistically significant differences observed compared to baseline measurements (Figure 3) (NRS score at baseline and at 6 months follow-up: 5.4 ± 1.9 vs. 1.7 ± 2.5, *p* < 0.05). The most substantial improvement was noted at the 6-month follow-up assessment (WOMAC total score at baseline and at 6 months follow-up: 86.1 ± 47.4 vs. 28.1 ± 46.9, *p* < 0.05). Knee functionality, evaluated using the WOMAC scale, exhibited a correlation with pain reduction, particularly evident in the pain and function subscales. However, regarding stiffness, statistically significant improvement compared to baseline was only evident at the 6-month mark, with no notable changes observed in earlier follow-up evaluations (Figure 4).

Additionally, a significative proportion of patients achieved the minimum clinically detectable improvement both in NRS and in WOMAC after the injections and specifically 79% for NRS (19 out of 24 patients) and 83% for WOMAC (20 out of 24 patients).

Notably, no patient complained about acute pain after injection.

### Correlation of baseline assessment and response rate

The K-L grade (5/14, 35.7% responders vs. 0/7, non-responders had a grade 2, 3/14, 21.4% responders vs. 2/7, 28.6% non-responders had a grade 3, 6/14, 42.9% responders vs. 5/7, 71.4% non-responders had a grade 4), the lack of response to previous treatments (6/19, 31.6% in responders vs. 1/5, 80.0% in non-responders) and US findings indicative of osteoarthritis (e.g., meniscal protrusion (6/19, 42.9% in responders vs. 1/5, 20.0% in non-responders), osteophytes (12/19, 63.2% in responders vs. 1/5, 20.0% in non-responders), and cartilage damage (12/19, 63.2% in responders vs. 1/5, 20.0% in non-responders) were numerically associated with a lack of response to HA treatment. However, their 95% CI is crossed the unit and none of them were significant predictors of NRS treatment response at univariable analyses.

### Safety

In our cohort we did not record any side effects related to injections. Specifically, no acute post-injection flare was reported.

## Discussion

Our data showed a significative efficacy of HMWHA-CTP injected under US guidance into the knees of patients with KOA and CPPD.

Osteoarthritis with CPPD is well known to be a more aggressive phenotype with more sustained and destructive inflammation (33); co-existence of calcium pyrophosphate crystals leads to a faster cartilage degradation and joint destruction. This consideration can, at least partially, explain the mean high radiographic OA grade in the patients of our cohort.

Moreover, in OA with CPPD, there was a persistent low grade inflammation that could be detected not only during the acute phase but also in the intercritical periods. Filippou et al. (34) described an increased US detectable s SH and joint effusion in CPPD-OA patients, that showed correlation with the number of CPP crystals in the synovial fluid but not with the number of polymorphonucleate cells, so excluding the contribute of an acute inflammation.

Given the inflammatory nature of CPPD associated OA, it is reasonable to hypothesize a role of HA and CTP as injective therapy.

High molecular weight hyaluronic acid has shown to carry out its action in OA mainly through an anti-inflammatory mechanism involving the suppression of several pro-inflammatory cytokines, disintegrines, and metalloproteinases and the induction of the synthesis of anti-inflammatory molecules. This happens by a binding of HMWHA to specific synovial receptors. On the contrary it seems that low molecular weight HA has more a pro-inflammatory effect (32).

Collagen tripeptide has shown an *in vitro* anti-inflammatory activity by inhibiting IL-1β and TNF-α production and upregulating IL-10 levels in synoviocytes cultures (14). The same authors, confirmed clinically the effect of collagen in OA by showing a significant reduction of Lequesne index, WOMAC index and VAS pain in patients treated with biweekly knee CTP injections for 6 months in respect to matched controls undergoing placebo (13).

Few years later another paper from the same group evaluated the effect of intraarticular knee injections of CTP after arthroscopic lavage on 19 OA patients in respect to placebo with a 6-months follow-up. Patients treated with CTP had improvement of Lequesne index, VAS, ESR, Tregs IL-1β, and IL-10 peripheral-expressing cells, concluding that CTP is effective on downregulating inflammation in KOA (15).

It is reasonable to consider that the combination of HMWHA and CTP can provide a strong anti-inflammatory effect, being potentially effective in OA with CPPD.

Currently, literature data about viscosupplementation in patients with KOA and CPPD are restricted to very few cases, some of them pointing toward the risk of having side effects more than benefits. Specifically there are three case reports of pseudogout attack following hyaluronate injection, one of them in a patient not known with CPPD before the injection, with the diagnosis being confirmed only after microscope analysis of the fluid (32, 35, 36).

The only data about the efficacy of intraarticular knee injection with HA in patient with OA and CPPD are restricted to small case series and are pointing toward good results (3).

Our data show a very good response to intra-articular HMWHA-CTP injections, with clinically relevant results as early as after 1 month and a progressive improvement through subsequent follow-up until 6 months. Notably, we are describing the largest cohort of KOA-CPPD treated with HA intra-articular injection by now and the first attempt ever with HMWHA-CTP. These results are promising and a longer follow-up in a larger cohort of patients will be necessary to define the timing for a second injection due to loss of efficacy. A larger cohort is also necessary to detect and describe infrequent side effects.

The analysis on baseline conditions (both clinical and US) did not show any correlation with positive or negative response to treatment. This can be due to the small number of patients. Some variables showed a tendency to be statistically significant, but the most interesting conclusion that we can get from the study is that viscosupplementation with HMWHA-CTP is working in KOA with CPPD independently on demographic data, OA radiological grade and baseline inflammation. Notably, in our population, we had a high proportion of patients with Kellgren-Lawrence grade 4 OA; usually these patients are excluded from studies on viscosupplementation being considered for surgical management. In our case, also these patients have obtained good results from intraarticular injections and this could be explained by the fact that in end-stage OA, synovium displays a grade of inflammation higher than in early phases (37).

## Conclusion

In conclusion, intra-articular HMWHA-CTP seems an effective and safe therapy in patients with KOA and CPPD.

## Data availability statement

The raw data supporting the conclusions of this article will be made available by the authors, without undue reservation.

## Ethics statement

The studies involving humans were approved by the Local Ethics Committee of ECH Craiova (No. 22763/09.05.2023). The studies were conducted in accordance with the local legislation and institutional requirements. The participants provided their written informed consent to participate in this study. Written informed consent was obtained from the individual(s) for the publication of any potentially identifiable images or data included in this article.

## Author contributions

FP: Conceptualization, Investigation, Methodology, Project administration, Resources, Supervision, Writing – original draft, Writing – review & editing. EF: Methodology, Supervision, Validation, Writing – original draft, Writing – review & editing. EC: Data curation, Methodology, Writing – original draft, Writing – review & editing. ML: Methodology, Writing – original draft, Writing – review & editing. XB: Data curation, Writing – original draft, Writing – review & editing. SS: Writing – original draft, Writing – review & editing. FV: Conceptualization, Data curation, Investigation, Methodology, Resources, Writing – original draft, Writing – review & editing.

## References

[1] Pereira D Peleteiro B Araújo J Branco J Santos RA Ramos E. The effect of osteoarthritis definition on prevalence and incidence estimates: A systematic review. *Osteoarthritis Cartilage.* (2011) 19:1270–85. 10.1016/j.joca.2011.08.009h 21907813

[2] McAlindon TE Bannuru RR Sullivan MC Arden NK Berenbaum F Bierma-Zeinstra SM OARSI guidelines for the non-surgical management of knee osteoarthritis. *Osteoarthritis Cartilage.* (2014) 22:363–88. 10.1016/j.joca.2014.01.003 24462672

[3] Voulgari PV Venetsanopoulou AI Drosos AA. Recent advances in the therapeutic management of calcium pyrophosphate deposition disease. *Front Med.* (2024) 11:1327715. 10.3389/fmed.2024.1327715 38529115 PMC10961350

[4] Stack J McCarthy G. Calcium pyrophosphate deposition (CPPD) disease – Treatment options. *Best Pract Res Clin Rheumatol.* (2021) 35:101720. 10.1016/j.berh.2021.101720 34756508

[5] Damart J Filippou G Andrès M Cipolletta E Sirotti S Carboni D Retention, safety and efficacy of off-label conventional treatments and biologics for chronic calcium pyrophosphate crystal inflammatory arthritis. *Rheumatology.* (2024) 63:446–55. 10.1093/rheumatology/kead228 37216917

[6] Guo S Lee C Wise B. Chondrocalcinosis and osteoarthritis: A literature review. *Eur J Rheumatol*. (2024) 11:15–20. 10.5152/eurjrheum.2023.21093 36688798 PMC11664833

[7] Foreman SC Gersing AS von Schacky CE Joseph GB Neumann J Lane NE Chondrocalcinosis is associated with increased knee joint degeneration over 4 years: Data from the osteoarthritis initiative. *Osteoarthritis Cartilage.* (2020) 28:201–7. 10.1016/j.joca.2019.10.003 31629813 PMC7002267

[8] Gersing AS Schwaiger BJ Heilmeier U Joseph GB Facchetti L Kretzschmar M Evaluation of chondrocalcinosis and associated knee joint degeneration using MR imaging: Data from the osteoarthritis initiative. *Eur Radiol.* (2017) 27:2497–506. 10.1007/s00330-016-4608-8 27704199

[9] Cipolletta E Francioso F Smerilli G Di Battista J Filippucci E. Ultrasound reveals a high prevalence of CPPD in consecutive patients with knee pain. *Clin Rheumatol.* (2024) 43:435–41. 10.1007/s10067-023-06805-3 37975949

[10] Wilkins E Dieppe P Maddison P Evison G. Osteoarthritis and articular chondrocalcinosis in the elderly. *Ann Rheum Dis.* (1983) 42:280–4. 10.1136/ard.42.3.280 6859960 PMC1001132

[11] Cooper C Rannou F Richette P Bruyère O Al-Daghri N Altman RD Use of intraarticular hyaluronic acid in the management of knee osteoarthritis in clinical practice. *Arthritis Care Res.* (2017) 69:1287–96. 10.1002/acr.23204 28118523 PMC5432045

[12] Bahrami MH Raeissadat SA Cheraghi M Rahimi-Dehgolan S Ebrahimpour A. Efficacy of single high-molecular-weight versus triple low-molecular-weight hyaluronic acid intra-articular injection among knee osteoarthritis patients. *BMC Musculoskelet Disord.* (2020) 21:550. 10.1186/s12891-020-03577-8 32799851 PMC7429877

[13] Furuzawa-Carballeda J Muñoz-Chablé O Macías-Hernández S Agualimpia-Janning A. Effect of polymerized-type I collagen in knee osteoarthritis. II. In vivo study. *Eur J Clin Invest.* (2009) 39:598–606. 10.1111/j.1365-2362.2009.02144.x 19397687

[14] Furuzawa-Carballeda J Muñoz-Chablé OA Barrios-Payán J Hernández-Pando R. Effect of polymerized-type I collagen in knee osteoarthritis. I. In vitro study. *Eur J Clin Invest.* (2009) 39:591–7. 10.1111/j.1365-2362.2009.02154.x 19453649

[15] Furuzawa-Carballeda J Lima G Llorente L Nuñez-Álvarez C Ruiz-Ordaz BH Echevarría-Zuno S. Polymerized-type I collagen downregulates inflammation and improves clinical outcomes in patients with symptomatic knee osteoarthritis following arthroscopic lavage: A randomized, double-blind, and placebo-controlled clinical trial. *ScientificWorldJournal.* (2012) 2012:342854. 10.1100/2012/342854 22545014 PMC3322395

[16] Naraoka T Ishibashi Y Tsuda E Yamamoto Y Kusumi T Toh S. Periodic knee injections of collagen tripeptide delay cartilage degeneration in rabbit experimental osteoarthritis. *Arthritis Res Ther.* (2013) 15:R32. 10.1186/ar4181 23433227 PMC3672813

[17] Xu Q Torres JE Hakim M Babiak PM Pal P Battistoni CM Collagen- and hyaluronic acid-based hydrogels and their biomedical applications. *Mater Sci Eng R Rep.* (2021) 146:100641. 10.1016/j.mser.2021.100641 34483486 PMC8409465

[18] Zhang W Doherty M Bardin T Barskova V Guerne PA Jansen TL European league against rheumatism recommendations for calcium pyrophosphate deposition. Part I: Terminology and diagnosis. *Ann Rheum Dis.* (2011) 70:563–70. 10.1136/ard.2010.139105 21216817

[19] Sirotti S Becce F Sconfienza LM Terslev L Naredo E Zufferey P Reliability and diagnostic accuracy of radiography for the diagnosis of calcium pyrophosphate deposition: Performance of the novel definitions developed by an international multidisciplinary working group. *Arthritis Rheumatol.* (2023) 75:630–8. 10.1002/art.42368 36122187

[20] Filippou G Scirè CA Damjanov N Adinolfi A Carrara G Picerno V Definition and reliability assessment of elementary ultrasonographic findings in calcium pyrophosphate deposition disease: A study by the OMERACT calcium pyrophosphate deposition disease ultrasound subtask force. *J Rheumatol.* (2017) 44:1744–9. 10.3899/jrheum.161057 28250136

[21] Bruyn GA Iagnocco A Naredo E Balint PV Gutierrez M Hammer HB OMERACT definitions for ultrasonographic pathologies and elementary lesions of rheumatic disorders 15 years on. *J Rheumatol.* (2019) 46:1388–93. 10.3899/jrheum.181095 30709946

[22] Bruyn GA Naredo E Damjanov N Bachta A Baudoin P Hammer HB An OMERACT reliability exercise of inflammatory and structural abnormalities in patients with knee osteoarthritis using ultrasound assessment. *Ann Rheum Dis.* (2016) 75:842–6. 10.1136/annrheumdis-2014-206774 25902788

[23] Möller I Janta I Backhaus M Ohrndorf S Bong DA Martinoli C The 2017 EULAR standardised procedures for ultrasound imaging in rheumatology. *Ann Rheum Dis.* (2017) 76:1974–9. 10.1136/annrheumdis-2017-211585 28814430

[24] Hartung W Kellner H Strunk J Sattler H Schmidt WA Ehrenstein B Development and evaluation of a novel ultrasound score for large joints in rheumatoid arthritis: One year of experience in daily clinical practice. *Arthritis Care Res.* (2012) 64:675–82. 10.1002/acr.21574 22183834

[25] Filippou G Scirè CA Adinolfi A Damjanov NS Carrara G Bruyn GAW Identification of calcium pyrophosphate deposition disease (CPPD) by ultrasound: Reliability of the OMERACT definitions in an extended set of joints - An international multiobserver study by the OMERACT calcium pyrophosphate deposition disease ultrasound subtask force. *Ann Rheum Dis.* (2018) 77:1195–200. 10.1136/annrheumdis-2017-212542 29535120

[26] Muzaffar N Kirmani O Ahsan M Ahmad S. Meniscal extrusion in the knee: Should only 3 mm extrusion be considered significant? An assessment by MRI and arthroscopy. *Malays Orthop J.* (2015) 9:17–20. 10.5704/MOJ.1507.013 28435604 PMC5333659

[27] Chagas-Neto FA Taneja AK Gregio-Junior E Nogueira-Barbosa MH. In-plane ultrasound-guided knee injection through a lateral suprapatellar approach: A safe technique. *Ultrasound Q.* (2017) 33:139–43. 10.1097/RUQ.0000000000000288 28481763

[28] Williamson A Hoggart B. Pain: A review of three commonly used pain rating scales. *J Clin Nurs.* (2005) 14:798–804. 10.1111/j.1365-2702.2005.01121.x 16000093

[29] Conaghan PG Dworkin RH Schnitzer TJ Berenbaum F Bushmakin AG Cappelleri JC WOMAC meaningful within-patient change: Results from 3 studies of tanezumab in patients with moderate-to-severe osteoarthritis of the hip or knee. *J Rheumatol.* (2022) 49:615–21. 10.3899/jrheum.210543 35232805

[30] Salaffi F Stancati A Silvestri CA Ciapetti A Grassi W. Minimal clinically important changes in chronic musculoskeletal pain intensity measured on a numerical rating scale. *Eur J Pain.* (2004) 8:283–91. 10.1016/j.ejpain.2003.09.004 15207508

[31] Clement ND Bardgett M Weir D Holland J Gerrand C Deehan DJ. What is the minimum clinically important difference for the WOMAC index after TKA? *Clin Orthop.* (2018) 476:2005–14. 10.1097/CORR.0000000000000444 30179956 PMC6259858

[32] Nicholls MA Fierlinger A Niazi F Bhandari M. The disease-modifying effects of hyaluronan in the osteoarthritic disease state. *Clin Med Insights Arthritis Musculoskelet Disord.* (2017) 10:117954411772361. 10.1177/1179544117723611 28839448 PMC5555499

[33] Stücker S Bollmann M Garbers C Bertrand J. The role of calcium crystals and their effect on osteoarthritis pathogenesis. *Best Pract Res Clin Rheumatol.* (2021) 35:101722. 10.1016/j.berh.2021.101722 34732285

[34] Filippou G Scanu A Adinolfi A Picerno V Toscano C Bortoluzzi A The two faces of the same medal…or maybe not? Comparing osteoarthritis and calcium pyrophosphate deposition disease: A laboratory and ultrasonographic study. *Clin Exp Rheumatol.* (2021) 7:66–72. 10.55563/clinexprheumatol/gu9j0q 32301428

[35] Luzar MJ Altawil B. Pseudogout following intraarticular injection of sodium hyaluronate. *Arthritis Rheum.* (1998) 41:939–40. 10.1002/1529-0131(199805)41:53.0.CO;2-D 9588748

[36] Maillefert JF Hirschhorn F Pascaud C Piroth A Tavernier C. Acute attack of chondrocalcinosis after an intraarticular injection of hyaluronan. *Rev Rhum Engl Ed.* (1997) 64:593–4. 9385702

[37] Ene Z Sinescu RD Ene P Cîrstoiu MM. Synovial inflammation in patients with different stages of knee osteoarthritis. *Rom J Morphol Embryol.* (2015) 56:169–73. 25826502

